# Synopsis of *Trichosanthes* (Cucurbitaceae) based on recent molecular phylogenetic data

**DOI:** 10.3897/phytokeys.12.2952

**Published:** 2012-04-19

**Authors:** Hugo J. de Boer, Mats Thulin

**Affiliations:** 1 Department of Systematic Biology, Uppsala University, Norbyvägen 18 D, SE-75236 Uppsala, Sweden

**Keywords:** Cucurbitaceae, *Trichosanthes*, *Gymnopetalum*, infrageneric classification, new combinations

## Abstract

The snake gourd genus, *Trichosanthes*, is the largest genus in the Cucurbitaceae family, with over 90 species. Recent molecular phylogenetic data have indicated that the genus *Gymnopetalum* is to be merged with *Trichosanthes* to maintain monophyly. A revised infrageneric classification of *Trichosanthes* including *Gymnopetalum* is proposed with two subgenera, (I) subg. *Scotanthus* comb. nov. and (II) subg. *Trichosanthes*, eleven sections, (i) sect. *Asterospermae*, (ii) sect. *Cucumeroides*, (iii) sect. *Edulis*, (iv) sect. *Foliobracteola*, (v) sect. *Gymnopetalum*, (vi) sect. *Involucraria*, (vii) sect. *Pseudovariifera* sect. nov., (viii) sect. *Villosae* stat. nov., (ix) sect. *Trichosanthes*, (x) sect. *Tripodanthera*, and (xi) sect. *Truncata*. A synopsis of *Trichosanthes* with the 91 species recognized here is presented, including four new combinations, *Trichosanthes orientalis*, *Trichosanthes tubiflora*, *Trichosanthes scabra* var. *pectinata*, *Trichosanthes scabra* var. *penicaudii*, and a clarified nomenclature of *Trichosanthes costata* and *Trichosanthes scabra*.

## Introduction

### Background

*Trichosanthes* L. is the largest genus in the Cucurbitaceae family, with over 90 species. The genus has its center of diversity in Southeast Asia, but ranges from India throughout Asia east to Taiwan, the Philippines and Japan, and southeast to New Guinea, Australia, Fiji and Vanuatu (de Wilde and Duyfjes 2010). The snake gourd (*Trichosanthes cucumerina* L.) is a popular vegetable in South and Southeast Asian cuisine and cultivated in tropical and subtropical regions around the globe. *Gymnopetalum* Arn. includes four species (de Wilde and Duyfjes 2006) and ranges from India through China and Southeast Asia into the Malay archipelago, but does not occur in New Guinea and Australia (de Wilde and Duyfjes 2010).

### Morphology and classification

*Trichosanthes* are mostly stout perennial climbers, 3–30 m long, dioecious, less frequently monoecious, with branched tendrils, distinctly fringed petals, and often egg-sized brightly colored fruits. Dioecy, variation in vegetative morphology (esp. in juvenile plants), and incomplete herbarium collections, complicate taxonomical studies and have contributed to the description of nearly 300 taxa (de Wilde and Duyfjes 2010; IPNI 2011).

No full taxonomic treatment of the genus exists, but in recent years regional revisions have been published for most of its distribution: India (Chakravarty 1959; Jeffrey 1980a; Jeffrey 1982), China (Lu et al. 2011; Yueh and Cheng 1974; Yueh and Cheng 1980), Thailand (Duyfjes and Pruesapan 2004), Cambodia, Laos and Vietnam (Keraudren-Aymonin 1975), Malaysia, Indonesia, the Philippines and Papua New Guinea (Rugayah and de Wilde 1997; Rugayah and de Wilde 1999; de Wilde and Duyfjes 2004; de Wilde and Duyfjes 2010), Australia (Telford 1982; Cooper and de Boer 2011) and Japan (Ohba 1984).

Infrageneric classifications of the genus *Trichosanthes* have been proposed by various authors (Yueh and Cheng 1974; Jeffrey 1980b; Chen 1985; Huang et al. 1997; Rugayah and de Wilde 1999; de Wilde and Duyfjes 2004). The most recent classifications of the genus (Rugayah and de Wilde 1999; de Wilde and Duyfjes 2004) propose six sections: (i) sect. *Trichosanthes*, (ii) sect. *Cucumeroides* (Gaertn.) Kitam. including subsect. *Cucumeroides* (Gaertn.) Kitam. and subsect. *Tetragonosperma* (C.Y.Cheng & Yueh) Rugayah, (iii) sect. *Edulis* Rugayah, (iv) sect. *Foliobracteola* C.Y.Cheng & Yueh, (v) sect. *Involucraria* (Ser.) Wight including subsect. *Bracteatae* C.Jeffrey ex S.K.Chen and subsect. *Pedatae* (C.Y.Cheng & Yueh) C.Jeffrey ex S.K.Chen, and (vi) sect. *Asterosperma* W.J.de Wilde & Duyfjes. However, both Rugayah and de Wilde (1999) and de Wilde and Duyfjes (2004; 2010) are reserved in their infrageneric classifications, and mention a need for further investigation.

Pollen morphology has also been used for infrageneric classification in the genus (Khunwasi 1998; Pruesapan and Van Der Ham 2005; Huang et al. 1997), but as a character is very variable in Cucurbitaceae, and its taxonomic value is not clear (Schaefer and Renner 2011). Palynological studies (Pruesapan and Van Der Ham 2005) have indicated that a variety of pollen types exist in *Trichosanthes* including 3(-4)-porate and 3(-4)-colporate pollen with psilate, perforate, verrucate, reticulate, and regulate ornamentation. Their study of pollen from 37 species distinguishes five pollen types, two of which are further divided into subtypes, and categorized these using exine ornamentation patterns for the major types and ectoaperture characters for the subtypes.

*Gymnopetalum* strongly resembles certain *Trichosanthes* species, but lack thread-like fringes on the petals, and the overall shape of the folded petals in the mature bud is elongate (short and rounded in *Trichosanthes*) (de Wilde and Duyfjes 2006). A revision of the genus was published by de Wilde and Dufyjes (2006), with minor nomenclatural changes published later (de Wilde and Duyfjes 2008). Cogniaux (1881) divided *Gymnopetalum* into two sections, (i) sect. *Gymnopetalum* containing the type *Gymnopetalum tubiflorum* (Wight & Arn.) Cogn. from southern India and Sri Lanka, and (ii) sect. *Tripodanthera* (M.Roem.) Cogn. containing the Southeast Asian and Malesian species. Later authors did not follow this classification (Jeffrey 1980a; Philcox 1997; de Wilde and Duyfjes 2006).

### Recent molecular phylogenetic studies

The molecular phylogenetic study of *Trichosanthes* by de Boer et al. (Submitted) shows that *Trichosanthes* and *Gymnopetalum* are both non-monophyletic, but together form a clade with high support in the Bayesian tree and weak support in the ML tree (0.99/62). This indicates that *Gymnopetalum* should be merged with *Trichosanthes*, and that a revised infrageneric classification is necessary. Some previously recognized sections in *Trichosanthes* and *Gymnopetalum* are well supported, but others need to be described or redefined.

## Results and discussion

### Molecular phylogeny

The molecular phylogeny of *Trichosanthes* and *Gymnopetalum* by de Boer et al. (Submitted) has nomenclatural implications for the species in *Gymnopetalum* and the infrageneric classification of *Trichosanthes*. The species of *Gymnopetalum* are placed in different clades within *Trichosanthes*, with the sect. *Gymnopetalum*, including the type *Gymnopetalum tubiflorum*, grouping in a well-supported clade (1.00/84) together with species of sect. *Trichosanthes* and sect. *Cucumeroides*. The sect. *Tripodanthera*, consisting of the three other species in the genus, *Gymnopetalum orientale* W.J.de Wilde & Duyfjes, *Gymnopetalum chinense* (Lour.) Merr., and *Gymnopetalum scabrum* (Lour.) W.J.de Wilde & Duyfjes, forms a well-supported clade (1.00/86) together with the taxa in *Trichosanthes* sect. *Edulis*.

Within *Trichosanthes* the support for the two clades here defined as subgenera *Trichosanthes* (1.00/94) and *Scotanthus* (1.00/97) is high. However,splitting the genus into two genera corresponding to subg. *Scotanthus* and subg. *Trichosanthes* would not improve clarity, as both would consist of species with fringed and fringeless corollas. Maintaining a large *Trichosanthes* is in accordance with the recent taxonomic revisions of the genus (de Wilde and Duyfjes 2010; Chakravarty 1959; Lu et al. 2011; Duyfjes and Pruesapan 2004; Keraudren-Aymonin 1975; Cooper and de Boer 2011; Ohba 1984), and is the alternative that best provides taxonomic stability.

Some proposed sections in *Trichosanthes* and *Gymnopetalum* are well supported: (i) sect. *Cucumeroides* (1.00/93), including subsect. *Cucumeroides* (0.99/-) and subsect. *Tetragonosperma* (0.99/69), (ii) sect. *Edulis* (1.00/75), and (iii) sect. *Asterosperma* (1.00/100). The subsections of sect. *Cucumeroides* are statistically supported, but subsect. *Cucumeroides* consists solely of accessions of *Trichosanthes pilosa* Lour. and species that have been reduced to its synonymy (Cooper and de Boer 2011). Sect. *Involucraria* is only weakly supported (0.83/65), primarily due to the low support for inclusion of its type, *Trichosanthes wallichiana* (Ser.) Wight. The subsections of *Involucraria* are not supported, and taxa belonging to subsect. *Pedatae* are nested in different locations within subsect. *Bracteatae*. Sect. *Tripodanthera* is not supported by the analysis, and could possibly form a grade at the base of sect. *Edulis*. However, morphological support for this section is strong as all taxa share characters of flower morphology, i.e. fringeless corollas. Sect. *Foliobracteola* Cheng & Yueh, which in its original sense included the species related to *Trichosanthes kirilowii* Maxim. and *Trichosanthes villosa* Blume (Yueh and Cheng 1974; Rugayah and de Wilde 1999; de Wilde and Duyfjes 2004), is not supported. However, a clade consisting of *Trichosanthes kirilowii* Maxim., *Trichosanthes miyagii* Hayata, *Trichosanthes homophylla* Hayata, *Trichosanthes hylonoma* Hand.-Mazz., *Trichosanthes rosthornii* Harms, and *Trichosanthes multiloba* Miq. is strongly supported (1.00/99). Section *Truncata*, in its original sense including *Trichosanthes truncata* C.B.Clarke, *Trichosanthes kerrii* Craib, *Trichosanthes homophylla* Hayata (Yueh and Cheng 1980), and *Trichosanthes smilacifolia* C.Y.Wu (Jeffrey 1980b), is not supported, as the three latter species all end up elsewhere in the phylogenetic tree.

*Trichosanthes villosa* was placed in sect. *Involucraria* by Yueh and Cheng (1974), and later in subsect. *Involucraria* by Jeffrey (1980b), but subsequently moved to sect. *Foliobracteola* by Rugayah and de Wilde (1999). In the protologue of *Trichosanthes phonsenae* Duyfjes and Pruesapan (2004), the authors stated that the three species *Trichosanthes kerrii*, *Trichosanthes phonsenae* and *Trichosanthes villosa* form a coherent, distinct group based on presence of white fruit pulp, male flowers with the stamens inserted low in the receptacle tube, and a pseudo-ovary. The molecular evidence shows that all accessions of these species in this study form a well-supported monophyletic group, confirming the observations by Duyfjes and Pruesapan (2004) and warranting placement of these taxa in a new section, sect. *Pseudovariifera* H.J.de Boer.

Cooper and Ford (2010) and Cooper and de Boer (2011) placed *Trichosanthes subvelutina* F.Muell. ex Cogn. in section *Foliobracteola* as it has obovate seeds with an entire broad marginal band similar to those found in Malesian species of section *Foliobracteola*. However, Huang et al (1997) proposed to place it in section *Foliobracteola* subsect. *Villosae* based on it pollen morphology. The current phylogenetic data place the accessions of *Trichosanthes subvelutina* as sister (1.00/91) to a well-supported clade (1.00/84) consisting of sections *Gymnopetalum*, *Trichosanthes*, and *Cucumeroides*, and the species is here placed in a separate section, sect. *Villosae* (Yueh & L.Q.Huang) H.J.de Boer.

### Pollen

Species with colporate ectoaperturate pollen form two monophyletic groups in *Trichosanthes* subg. *Trichosanthes*, one including sections *Asterosperma*, *Pseudovariifera*, *Foliobracteola*, and *Truncata*, and the other including sect. *Villosae*, with *Trichosanthes subvelutina* (data from R. van der Ham 2011, pers. comm.). The latter clade is sister to the clade consisting of sections *Trichosanthes*, *Gymnopetalum* and *Cucumeroides* (1.00/84). The remaining sections in subg. *Trichosanthes* have porate ectoapertures, and varying exine ornamentation including psilate, (micro-)reticulate, perforate, verrucate, and rugulate pollen (Huang et al. 1997; Pruesapan and Van Der Ham 2005). *Gymnopetalum scabrum* in sect. *Tripodanthera* has 3-colporate, rugulate-reticulate pollen (Khunwasi 1998; van der Ham et al. 2010), whereas in *Gymnopetalum tubiflorum* in sect. *Gymnopetalum* the pollen is 3-porate and microreticulate (R. van der Ham 2010, pers. comm.).

The other genera in the tribe Sicyoeae have colpate-colporate pollen, similar to that in many other distantly related groups in Curcurbitaceae (Schaefer and Renner 2011). In the light of the molecular data and the phylogenetic analysis (de Boer et al. Submitted), a transition from colporate to porate apertures has taken place three times in the evolutionary history of *Trichosanthes*, in the common ancestors of: 1) sect. *Involucraria*; 2) sect. *Edulis*; and 3) sections *Trichosanthes*, *Gymnopetalum* and *Cucumeroides*.

## Taxonomy and classification

A revision of the infrageneric classifications suggested by previous authors on the basis of morphological studies (Yueh and Cheng 1974; Jeffrey 1980b; Huang et al. 1997; Rugayah and de Wilde 1999; de Wilde and Duyfjes 2004) is here proposed on the basis of the molecular phylogenetic data (de Boer et al. Submitted). A synopsis is presented in which we attempt to assign all 91 species recognized here to sections using the clades recovered in the phylogenetic analysis as a framework, along with a plenitude of data from macromorphological studies of herbarium vouchers (Chakravarty 1959; Jeffrey 1980a; Jeffrey 1980b; Jeffrey 1982; Telford 1982; Ohba 1984; Rugayah and de Wilde 1997; Rugayah and de Wilde 1999; Duyfjes and Pruesapan 2004; de Wilde and Duyfjes 2004; de Wilde and Duyfjes 2006; Cooper and Ford 2010; de Wilde and Duyfjes 2010; Lu et al. 2011; Cooper and de Boer 2011) and palynological work (Huang et al. 1997; Pruesapan and Van Der Ham 2005; van der Ham et al. 2010). Synonyms are only included if these are new or relevant for this paper. Names that have been placed in synonymy by previous authors can be found in the above-cited morphological studies.

***Trichosanthes*** L. (1753) Sp. Pl. 2: 1008 – Type: *Trichosanthes anguina* L. [= *Trichosanthes cucumerina* L.]

***Trichosanthes* subg. *Scotanthus*** (Kurz) H.J.de Boer, comb. nov. – *Gymnopetalum* subg. *Scotanthus* Kurz (1877) J. Asiat. Soc. Bengal, Pt. 2, Nat. Hist. 46: 99. – Lectotype, designated here: *Bryonia cochinchinensis* Lour. [ = *Trichosanthes costata* Blume]

*Scotanthus* Naud., nom. illeg. (1862) Ann. Sci. Nat., Bot. sér. 4, 16: 172. – Type: *Momordica tubiflora* Roxb. [ = *Trichosanthes costata* Blume]

***Trichosanthes* sect. *Edulis***Rugayah (1999) Reinwardtia 11: 232. – Type: *Trichosanthes edulis* Ruguyah.

*Trichosanthes densiflora* Rugayah (1999) Reinwardtia 11: 252

*Trichosanthes dentifera* Rugayah (1999) Reinwardtia 11: 253

*Trichosanthes dieniensis* Merr. & L.M.Perry (1949) J. Arnold Arbor. 30: 59

*Trichosanthes edulis* Rugayah (1999) Reinwardtia 11: 254

*Trichosanthes hastata* Harms (1925) Bot. Jahrb. Syst. 60: 160

*Trichosanthes laeoica* C.Y.Cheng & Lu Q.Huang (1996) Bull. Bot. Res., Harbin 16: 503

*Trichosanthes odontosperma* W.E.Cooper & A.J.Ford (2010) Austrobaileya 8: 126

*Trichosanthes pulleana* Harms (1925) Bot. Jahrb. Syst. 60: 160

*Trichosanthes schlechteri* Harms (1925) Bot. Jahrb. Syst. 60: 159

***Trichosanthes* sect. *Involucraria***(Ser.) Wight (1840) Madras J. Lit. Sci. 12: 52. – *Involucraria* Ser. (1825) Mém. Soc. Phys. Genève 3: 27, t. 5. – Type: *Involucraria wallichiana* Ser. [ = *Trichosanthes wallichiana* (Ser.) Wight]

*Trichosanthes anamalaiensis* Bedd. (1864) Madras J. Lit. Sci. III, 1: 47

*Trichosanthes borneensis* Cogn. (1881) Monogr. Phan. [A.DC. & C.DC.] 3: 369

*Trichosanthes bracteata* (Lam.) Voigt (1845) Hort. Suburb. Calcutt. 58

*Trichosanthes celebica* Cogn. (1881) Monogr. Phan. [A.DC. & C.DC.] 3: 385

*Trichosanthes cordata* Roxb. (1832) Fl. Ind. 3: 703

*Trichosanthes coriacea* Blume (1826) Bijdr. Fl. Ned. Ind. 15: 935

*Trichosanthes dolichosperma* Duyfjes & Pruesapan (2004) Thai Forest Bull., Bot. 32: 84

*Trichosanthes dunniana*H. Lév. (1911) Repert. Spec. Nov. Regni Veg. 10: 148

*Trichosanthes ellipsoidea* Merr. (1918) Philipp. J. Sci., C 13: 332

*Trichosanthes elmeri* Merr. (1929) Univ. Calif. Publ. Bot. 15: 299

*Trichosanthes emarginata* Rugayah (1999) Reinwardtia 11: 258

*Trichosanthes erosa*Duyfjes & Pruesapan (2004) Thai Forest Bull., Bot. 32: 85

*Trichosanthes fissibracteata* C.Y.Wu ex C.Y.Cheng & C.H.Yueh (1974) Acta Phytotax. Sin. 12: 438

*Trichosanthes floresana* Rugayah (1999) Reinwardtia 11: 260

*Trichosanthes globosa* Blume (1826) Bijdr. Fl. Ned. Ind. 15: 936

*Trichosanthes intermedia* W.J.de Wilde & Duyfjes (2004) Sandakania 14: 19

*Trichosanthes inthanonensis* Duyfjes & Pruesapan (2004) Thai Forest Bull., Bot. 32: 86

*Trichosanthes khasiana* Kundu (1939) J. Bot. 77: 11

*Trichosanthes kinabaluensis* Rugayah (2000) Reinwardtia 11: 419

*Trichosanthes kostermansii* Duyfjes & Pruesapan (2004) Thai Forest Bull., Bot. 32: 89

*Trichosanthes laceribractea* Hayata (1911) J. Coll. Sci. Imp. Univ. Tokyo 30. Art. 1: 117

*Trichosanthes lepiniana* (Naud.) Cogn. (1881) Monogr. Phan. [A.DC. & C.DC.] 3: 377

*Trichosanthes leuserensis* Rugayah (1999) Reinwardtia 11: 265

*Trichosanthes longispicata* Rugayah (1999) Reinwardtia 11: 266

*Trichosanthes montana* Rugayah (1998) Reinwardtia 11: 218

*Trichosanthes morrisii* W.E.Cooper (2011) Austrobaileya 8: 381

*Trichosanthes obscura* Rugayah (1999) Reinwardtia 11: 269

*Trichosanthes pallida*Duyfjes & Pruesapan (2004) Thai Forest Bull., Bot. 32: 90

*Trichosanthes papuana* F.M.Bailey (1900) Queensland Agric. J. 7: 349

*Trichosanthes pedata* Merr. & Chun (1934) Sunyatsenia 2: 20

*Trichosanthes pentaphylla* F.Muell. in Benth. (1867) Fl. Austral. 3: 314

*Trichosanthes philippinensis* Rugayah (1999) Reinwardtia 11: 271

*Trichosanthes planiglans* Rugayah (1999) Reinwardtia 11: 273

*Trichosanthes pubera* Blume (1826) Bijdr. Fl. Ned. Ind. 15: 936

*Trichosanthes quinquangulata* A. Gray (1854) U.S. Expl. Exped., Phan. 15: 645

*Trichosanthes quinquefolia* C.Y.Wu ex C.Y.Cheng & C.H.Yueh (1980) Acta Phytotax. Sin. 18: 351

*Trichosanthes refracta* C.H.Yueh (1996) Bull. Bot. Res., Harbin 10: 500

*Trichosanthes rugatisemina* C.Y.Cheng & C.H.Yueh (1974) Acta Phytotax. Sin. 12: 440

*Trichosanthes sepilokensis* Rugayah (1999) Reinwardtia 11: 275

*Trichosanthes subrosea* C.Y.Cheng & C.H.Yueh (1980) Acta Phytotax. Sin. 18: 349

*Trichosanthes tricuspidata* Lour. (1790) Fl. Cochinch. 2: 589

*Trichosanthes valida* Rugayah (1999) Reinwardtia 11: 277

*Trichosanthes wallichiana* (Ser.) Wight (1840) Madras J. Lit. Sci. 12: 52

*Trichosanthes wawrae* Cogn. (1881) Monogr. Phan. [A.DC. & C.DC.] 3: 384

***Trichosanthes* sect.** ***Tripodanthera*** (M.Roem.) H.J.de Boer, *comb. nov*. – *Tripodanthera* M.Roem. (1846) Fam. Nat. Syn. Monogr. 2: 48. – *Gymnopetalum* sect. *Tripodanthera* (M.Roem.) Cogn. (1881) Monogr. Phan. [A.DC. & C.DC.] 3: 390. – Type: *Bryonia cochinchinensis* Lour. [ = *Trichosanthes costata* Blume]

***Trichosanthes costata*** Blume (1826) Bijdr. Fl. Ned. Ind. 15: 933. — Type: *Blume s.n*. barcode L0589632, (lectotype L, designated by de Wilde and Duyfjes (2006); 2 isotypes L), Java, Indonesia. Heterotypic synonyms: *Evonymus chinensis* Lour. (1790) Fl. Cochinch. 1: 156. – *Gymnopetalum chinense* (Lour.) Merr. (1919) Philipp. J. Sci. 15: 256 – Type: *Loureiro* †. *Bryonia cochinchinensis* Lour. (1790) Fl. Cochinch. 1: 595. – *Gymnopetalum cochinchinense* (Lour.) Kurz (1871) J. Asiat. Soc. Bengal, Pt. 2, Nat. Hist. 40: 57 –Type: *Loureiro s.n.* (BM), Vietnam. *Momordica tubiflora* Roxb. (1832) Fl. Ind. 3: 711 – Type: *Wallich Cat. 6749* (holotype? K-W), Dacca, Bangladesh. Note: The existence of *Trichosanthes chinensis* Ser. (1828) Prodr. [A.P. de Candolle] 3: 315 blocks the transfer of *Gymnopetalum chinense*, based on the basionym *Evonymus chinensis*, to *Trichosanthes*. The second name in line of priority would be *Gymnopetalum cochinchinensis*, based on the basionym *Bryonia cochinchinensis*. However, the combination *Trichosanthes cochinchinensis* M.Roem. (1846) Fam. Nat. Syn. Monogr. 2: 96, based on *Trichosanthes cucumerina* Lour. (1790) 722 (non L.), blocks the transfer. The third name in line of priority is *Trichosanthes costata* Blume (1826) Bijdr. Fl. Ned. Ind. 15: 933, and this name is available for *Gymnopetalum chinense*.

***Trichosanthes scabra*** Lour. (1790) Fl. Cochinch. 2: 589. – *Gymnopetalum scabrum* (Lour.) W.J.de Wilde & Duyfjes (2008) Reinwardtia 12: 268. – Type: *Poilane 11322* (neotype P; isoneotype L, designated by de Wilde and Duyfjes (2008)), Annam. Heterotypic synonym: *Cucumis integrifolius* (‘*integrifolia*’) Roxb. (1832) Fl. Ind. 3: 724. – *Gymnopetalum integrifolium* (Roxb.) Kurz (1871) J. Asiat. Soc. Bengal, Pt. 2, Nat. Hist. 40: 58. –Type: *Wallich Cat. 6730* (holotype? K-W), Burma.

var. *scabra*

var. ***pectinata*** (W.J.de Wilde & Duyfjes) H.J.de Boer, *comb. nov*. – *Gymnopetalum scabrum* (Lour.) W.J.de Wilde & Duyfjes var. *pectinatum* (W.J. de Wilde & Duyfjes) W.J.de Wilde & Duyfjes (2008) Reinwardtia 12: 268 – *Gymnopetalum integrifolium* (Roxb.) Kurz var. *pectinatum* W.J.de Wilde & Duyfjes (2006), Blumea 51: 287. – Type: *W.J. de Wilde and Duyfjes 21692* (holotype L), Java, Indonesia.

var. ***penicaudii*** (Gagnep.) H.J.de Boer, *comb. nov*. – *Gymnopetalum penicaudii* Gagnep. (1918) Bull. Mus. Natl. Hist. Nat. 24: 374. – *Gymnopetalum scabrum* (Lour.) W.J.de Wilde & Duyfjes var. *penicaudii* (Gagnep.) W.J.de Wilde & Duyfjes (2008) Reinwardtia 12: 268 – Type: *Pénicaud 43* (lectotype P), Hainan, China.

***Trichosanthes orientalis*** (W.J.de Wilde & Duyfjes) H.J.de Boer, *comb. nov*. – *Gymnopetalum orientale*W.J.de Wilde & Duyfjes (2006) Blumea 51: 290. – Type: *De Wilde and Duyfjes 21937* (holotype L), Lombok, Indonesia.

***Trichosanthes* subg. *Trichosanthes***

Unresolved placement within this subgenus:

*Trichosanthes reticulinervis* C.Y.Wu ex S.K.Chen (1985) Bull. Bot. Res., Harbin 5(2): 114

*Trichosanthes smilacifolia* C.Y.Wu ex C.H.Yueh & C.Y.Cheng (1980) Acta Phytotax. Sin. 18: 347

***Trichosanthes* sect. *Asterosperma*** W.J.de Wilde & Duyfjes (2004) Sandakania 14: 6. – Type: *Trichosanthes postarii* W.J.de Wilde & Duyfjes.

*Trichosanthes auriculata* Rugayah (1998) Reinwardtia 11: 216

*Trichosanthes fusca* W.J.de Wilde & Duyfjes (2004) Sandakania 14: 17

*Trichosanthes postarii* W.J.de Wilde & Duyfjes (2004) Sandakania 14: 26

*Trichosanthes rotundifolia* Rugayah (1998) Reinwardtia 11: 223

***Trichosanthes* sect. *Cucumeroides*** (Gaertn.) Kitam. (1943) J. Jap. Bot. 19: 35. – *Cucumeroides* Gaertn. (1791) Fruct. Sem. Pl. 2: 485, t. 180, t. 4. – Type: *Trichosanthes cucumeroides* (Ser.) Maxim. [ = *Trichosanthes pilosa* Lour.].

*Trichosanthes adhaerens* W.J.de Wilde & Duyfjes (2004) Sandakania 14: 11

*Trichosanthes beccariana* Cogn. (1881) Monogr. Phan. [A.DC. & C.DC.] 3: 380

*Trichosanthes mucronata* Rugayah (1999) Reinwardtia 11: 268

*Trichosanthes pendula* Rugayah (1998) Reinwardtia 11 (3): 219

*Trichosanthes pilosa* Lour. (1790) Fl. Cochinch. 2: 588. Heterotypic synonyms according to Cooper and de Boer (2011): *Trichosanthes baviensis* Gagnep. (1918) Bull. Mus. Natl. Hist. Nat. 24: 379; *Trichosanthes trichocarpa* C.Y.Wu ex C.Y.Cheng & C.H.Yueh (1980) Acta Phytotax. Sin. 18: 340; *Trichosanthes holtzei* F.Muell. (1886) Australas. Journ. Pharm. 1: 447.

*Trichosanthes siamensis* Duyfjes & Pruesapan (2004) Thai Forest Bull., Bot. 32: 97

*Trichosanthes tetragonosperma* C.Y.Cheng & C.H.Yueh (1974) Acta Phytotax. Sin. 12: 425

***Trichosanthes* sect. *Foliobracteola*** C.Y.Cheng & C.H.Yueh (1974) Acta Phytotax. Sin. 12: 427. – Type: *Trichosanthes kirilowii* Maxim.

*Trichosanthes homophylla* Hayata (1921) Icon. Pl. Formosan. 10: 8

*Trichosanthes hylonoma* Hand.-Mazz. (1936) Symb. Sin. Pt. 7: 1066

*Trichosanthes ishigakiensis* E.Walker (1971) J. Jap. Bot. 46: 71

*Trichosanthes jinggangshanica* C.H.Yueh (1980) Acta Phytotax. Sin. 18: 342

*Trichosanthes kirilowii* Maxim. (1859) Prim. Fl. Amur. 482

*Trichosanthes mianyangensis* C.H.Yueh & R.G.Liao (1992) Bull. Bot. Res., Harbin 2: 115

*Trichosanthes miyagii* Hayata (1921) Icon. Pl. Formosan. 10: 11

*Trichosanthes multiloba* Miq. (1865) Ann. Mus. Bot. Lugd.-Bat. 2: 82

*Trichosanthes rosthornii* Harms (1901) Bot. Jahrb. Syst. 29: 603

***Trichosanthes* sect. *Gymnopetalum*** (Arn.) H.J.de Boer, *comb. et stat. nov*. – *Gymnopetalum* Arn. (1840) Madras J. Lit. Sci. 12: 52. –Type: *Bryonia tubiflora* Wight & Arn. [ = *Trichosanthes tubiflora* (Wight & Arn.) H.J.de Boer].

***Trichosanthes tubiflora*** (Wight & Arn.) H.J.de Boer, *comb. nov*. – *Bryonia tubiflora* Wight & Arn. (1834) Prodr. Fl. Ind. Orient. 1: 347. – *Gymnopetalum tubiflorum* (Wight & Arn.) Cogn. (1881) Monogr. Phan. [A.DC. & C.DC.] 3: 388 –Type: *Rottler s.n*. ex Herb. Klein in Herb. Wight Cat. 1118, February 1796 (holotype K; isotypes E, several duplicates), Trincomalee, Ceylon.

***Trichosanthes* sect. *Pseudovariifera***H.J.de Boer, *sect. nov*. Diagnosis: Similar to sect. *Foliobracteola*, but male flowers with stamens inserted low in receptacle tube, with pseudo-ovary (a thick-walled basal part of the receptacle tube, without staminodes), and fruit with white pulp. Type: *Trichosanthes villosa* Blume.

*Trichosanthes kerrii* Craib (1914) Bull. Misc. Inform. Kew: 7

*Trichosanthes phonsenae* Duyfjes & Pruesapan (2004) Thai Forest Bull., Bot. 32: 9

*Trichosanthes sericeifolia* C.Y.Cheng & C.H.Yueh (1980) Acta Phytotax. Sin. 18: 346

*Trichosanthes villosa* Blume (1826) Bijdr. Fl. Ned. Ind. 15: 934

***Trichosanthes* sect. *Trichosanthes***

*Trichosanthes cucumerina* L. (1753) Sp. Pl.: 1008

*Trichosanthes dafangensis* N.G.Ye & S.J.Li (1989) Acta Phytotax. Sin. 27: 153

*Trichosanthes dioica* Roxb. (1832) Fl. Ind. 3: 701

*Trichosanthes integrifolia* Thwaites (1859) Enum. Pl. Zeyl. [Thwaites]: 127

*Trichosanthes lobata* Roxb. (1832) Fl. Ind. 3: 703. – Heterotypic synonyms: *Trichosanthes perrotetiana* Cogn. (1881) Monogr. Phan. [A.DC. & C.DC.] 3: 362; *Trichosanthes villosula* Cogn. (1881) Monogr. Phan. [A.DC. & C.DC.] 3: 342

*Trichosanthes nervifolia* L. (1753) Sp. Pl.: 1008

***Trichosanthes* sect. *Truncata*** C.Y.Cheng & C.H.Yueh (1974) Acta Phytotax. Sin. 12: 427. – Type: *Trichosanthes truncata* C.B.Clarke

*Trichosanthes truncata* C.B.Clarke (1879) Fl. Brit. India [J.D. Hooker] 2: 608. – Heterotypic synonym: *Trichosanthes ovata* Cogn. (1881) Monogr. Phan. [A.DC. & C.DC.] 3: 365

***Trichosanthes* sect. *Villosae*** (Yueh & L.Q.Huang) H.J.de Boer, *stat. nov*. – *Trichosanthes* subsect. *Villosae* Yueh & L.Q.Huang (1997) Act. Phytotax. Sinica 35: 127. – Type: *Trichosanthes subvelutina* F.Muell. ex Cogn.

*Trichosanthes subvelutina* F.Muell. ex Cogn. (1881) Monogr. Phan. [A.DC. & C.DC.] 3: 366
